# Does adversity early in life affect general population suicide rates? a cross-national study

**DOI:** 10.5249/jivr.v3i1.70

**Published:** 2011-01

**Authors:** Ajit Shah, Ritesh Bhandarkar

**Affiliations:** ^*a*^University of Central Lancashire, Preston, United Kingdom and Consultant Psychiatrist, West London Mental Health NHS Trust, London, United Kingdom.; ^*b*^Trainee Psychiatirst, West London Mental Health NHS Trust, London, United Kingdom.

## Abstract

**Background:**

Adversity early in life has been suggested as a protective factor for elderly suicides. However, studies examining this relationship in general population suicide rates are scarce.

**Methods:**

The relationship between general population suicide rates and four proxy measures of adversity earlier in life was examined using data from the World Health Organization and the United Nations data banks.

**Results:**

General population suicide rates were negatively correlated with the percentage of children under the age of 5 years who were underweight, the percentage of children under the age of 5 years who were under height, the percentage of infants with low birth weight babies, and the percentage of the general population that was undernourished. The only independent predictor general population suicide rates in both sexes, on multiple regression analysis, was the Gini coefficient (a measure of income inequality).

**Conclusions:**

Income inequality may lead to low birth weight, undernourishment, underweight and under height because income inequality results in poor access to healthcare and nutrition. These adversities may increase child mortality rates and reduce life expectancy. Those surviving into adulthood in countries with greater adversity early in life may be at reduced risk of suicide because of selective survival of those at reduced risk of suicide due to constitutional or genetic factors and development of greater tolerance to hardship in adulthood.

## Introduction

Elderly suicide rates^1^and general population suicide rates^2^have been shown to be low in countries with low socio-economic status including those with greater income inequality. Life expectancy is also reduced in these countries.^1^Therefore, given that suicide rates generally increase with age,^3^is possible that in some countries with low elderly suicide rates fewer people will reach the age of increased risk of suicide due to reduced life expectancy. Moreover, selective survival of those at reduced risk for suicide due to genetic or constitutional factors may further compound this trend. Furthemore, those who do may be at reduced risk of suicide in old age because they may be able to tolerate extra hardship in old age better due to exposure to life-long adversity.^4,5^For example, elderly African Americans and native Americans (Indians) have low suicide rates^6^and this has been attributed to a life-long history of socio-economic deprivation.^6^The relationship between exposure to adversity early in life and general population suicide rates has not be examined. Therefore, a cross-national study examining the relationship between general population suicide rates and several proxy measures of adversity earlier in life was undertaken.

## Methods

Data on general population suicide rates for males and females was ascertained from the WHO website(www.who.int/whosis/database/mort/table1.cfm) for the latest available year, and the median (range) of this latest year across the different countries was 2000 (1985-2003). They cover all age groups.

The United Nations Development Programme (UNDP) website(www.hdr.undp.org/reports/global/2005/pdf/hdr05_HDI.pdf) provided data on the percentage of children under the age of 5 years who were underweight, the percentage of children under the age of 5 years who were underheight, the percentage of infants with low birth weight babies, and the percentage of the general population that was undernourished. These four variables were available for an unspecified year between 1996 and 2004. These four variables were considered to be proxy measures of adversity earlier in life; although these measures may have changed over time, they are likely to have improved and may still give an indication of adversity earlier in life. The relationship between general population suicide rates and the five proxy measures of adversity earlier in life was examined with Spearman’s rank correlation (rho).

The four measures of adversity earlier in life were inter-correlated. Moreover, previous studies have shown suicide rates to be correlated with GDP and income inequality measured by the Gini coefficient.^2,7-9^Thus, the independent relationship between general population suicide rates and measures of adversity earlier in life was examined using multiple regression analyses with the Enter method: general population suicide rate was the dependent variable and all four proxy measures of adversity earlier in life, GDP and the Gini coefficient were the independent variables. The WHO website (www.who.int/countries/en/) provided data on the GDP for the year 2002. The UNDP website provided data on a measure of income inequality called the Gini coefficient (www.hdr.undp.org/reports/global/2005/pdf/hdr05_HDI.pdf).

## Results

Table 1illustrates the relationship between general population suicide rates and the proxy measures of adversity earlier in life. Suicide rates in males and females were significantly and negatively correlated with the percentage of children under the age of 5 years who were underweight, the percentage of children under the age of 5 years who were underheight, the percentage of infants with low birth weight babies, and the percentage of the general population that was undernourished.

On multiple regression analyses, the only independent predictor of general population suicide rates in males (P=0.031) and females (P=0.009) was the Gini coefficient.

**Table 1:** The relationship between general population suicide rates and proxy measures of adversity earlier in life

	% Children Under Weight N*=44	% Children Under Height N*=46	% Infants With Low Birth N*=75	% of Total Population Undernourished Weight N*=52
Suicide Rates:				
Males	Rho=-0.38/P=0.011	Rho=-0.42/P=0.004	Rho=-0.38/P=0.001	Rho=-0.48/P<0.00001
Females	Rho=-0.25/P=0.097	Rho=-0.43/P=0.003	Rho=-0.36/P=0.002	Rho=-0.46/P<0.00001

N = The total number of countries with available data on the specific variable and suicide rates.

## Discussion

Some methodological issues need consideration. First, cross-national data on suicide rates should be viewed cautiously because: data are not available from all countries^10,11^; the validity of this data is unclear;^11,12^the legal criteria for the proof of suicide vary between countries and between different regions within a country;^11,13^some countries, particularly low-income countries, may have poor death registration facilities;^13^and, cultural and religious factors and stigma attached to suicide may lead to under-reporting of suicides.^11,14^Second, data on socio-economic status and proxy measures of adversity earlier in life should also be viewed cautiously because: the validity of this data is unclear; some countries may have poor registration facilities for data on these measures; and, some countries may have poor infrastructure for providing accurate financial data.^1^These latter concerns are more likely to be observed in low-income countries. Third, several variables were assumed to be proxy measures of adversity earlier in life. Fourth, caution should be exercised in assuming the direction of causal relationships in this cross-sectional and ecological study because of ecological fallacy. Nevertheless, the best and the latest available data were ascertained from the WHO and UNDP data banks.

The observed negative correlations between general population suicide rates and the four proxy measures of adversity earlier in life may be explained by the above methodological difficulties. However, it is possible that the correlations are genuine because they confirm earlier reports of an association between life-long adversity and lower elderly suicide rates.^4-6^Possible explanations for these correlations are explored below in the context of the finding from the multiple regression analyses that the Gini coefficient was the only independent predictor of general population suicide rates.

Income inequality, as measured by the Gini coefficient, may lead to low birth weight, undernourishment, underweight and underheight because income inequality results in poor access to healthcare and nutrition.^15^These adversities may increase child mortality rates. Increased child mortality rates, in turn, reduces life expectancy.^1,7,15^Given that suicide rates generally increase with age,^3,16^reduced life expectancy will result in fewer people reaching the age of increased risk for suicide (i.e. adulthood) in countries with greater adversity early in life. This is supported by positive correlations between life expectancy and suicide rates in the elderly^1^and the general population in cross-national studies. Suicide rates in those who survive into adulthood in such countries may be further reduced for two reasons. First, this trend may be further facilitated by selective survival of those at reduced risk for suicide due to genetic or constitutional factors. Second, those who survive into adulthood in such countries may be at reduced risk of suicide because they may have greater tolerance of additional hardship due to earlier exposure to adversity;^4,5^this has been suggested as an explanation for low elderly suicide rates among African Americans and native Americans in the US.^6^This hypothesis could be tested in long-term within-country longitudinal studies where adversity early in life changes over time.

## References

[1] Shah A, Bhat R, MacKenzie S, Koen C (2008). A cross-national study of the relationship between elderly suicide rates and life expectancy and markers of socioeconomic status and health care. Int Psychogeriatr.

[2] Vijayakumar L, Nagaraj K, Pirkis J, Whiteford H (2005). Suicide in developing countries (1): frequency, distribution, and association with socioeconomic indicators. Crisis.

[3] Shah A, De T (1998). Suicide and the elderly. Int J Psychiatry Clin Pract.

[4] Lindesay  J (1991). Suicide in the elderly. Int J Geriatric Psychiatry.

[5] Seiden RH (1981). Mellowing with age: factors affecting the non-white suicide rate. International Journal of Ageing and Human Development.

[6] McIntosh JL (1984). Components of the decline in elderly suicides: suicide in young old and old old by race and sex. Death Education.

[7] Zhang J (1998). Suicide in the world: toward a population increase theory of suicide. Death Stud.

[8] Cutright P, Fernquist RM (2000). Effects of social integration, period, region, and culture of suicide on male age-specific suicide rates: 20 developed countries, 1955-1898. Soc Sci Res.

[9] Gunnell D, Middleston N, Whitley E, Dorling D, Frankel S (2003). Why are suicide rates rising in young men but falling in the elderly? -- a time-series analysis of trends in England and Wales 1950-1998. Soc Sci Med.

[10] Moscicki EK (1995). North American perspectives: epidemiology of suicide. International Psychogeriatrics.

[11] Wasserman D, Cheng Q, Jiang GX (2005). Global suicide rates among young people aged 15-19. World Psychiatry.

[12] Diekstra RF (1989). Suicide and the attempted suicide: an international perspective. Acta Psychiatr Scand suppl.

[13] Shah A, Ganesvaran T. Suicide in the elderly. In: Chiu E, Ames D, (Eds): Functional Psychiatric Disorders of the Elderly. Cambridge: Cambridge University Press, 1994: 221-44.

[14] Abrahams VJ, Abrahams S, Jacob KS (2005). Suicide in the elderly in Kanyambadi block, Tamil Nadu, South India. Int J Geriatr Psychiatry.

[15] Shah A (2007). The importance of the socioeconomic status of countries for mental disorders in old age: development of an epidemiological transition model. Int Psychogeriatr.

[16] Shah A (2007). The relationship between suicide rate and age: an analysis of multinational data from the World Health Organisation. Int Psychogeriatr.

